# Prevalence and Molecular Characterization of the Zoonotic Enteric Protozoans *Cryptosporidium* spp., Enterocytozoon bieneusi, and *Blastocystis* from Pallas’s Squirrels (Callosciurus erythraeus) in Kanagawa Prefecture, Japan

**DOI:** 10.1128/Spectrum.00990-21

**Published:** 2021-11-03

**Authors:** Aya Masuda, Minami Wada, Haruka Saho, Kako Tokunaga, Yuto Kikuchi, Fumiaki Yamasaki, Jun Matsumoto

**Affiliations:** a Laboratory of Medical Zoology, Bioresource Sciences, Nihon University, Fujisawa, Kanagawa, Japan; b School of Veterinary Medicine, Nippon Veterinary and Life Science University, Tokyo, Japan; Hebrew University of Jerusalem

**Keywords:** Pallas's squirrels (*Callosciurus erythraeus*), *Cryptosporidium ubiquitum*, *Enterocytozoon bieneusi*, *Blastocystis*

## Abstract

Pallas’s squirrel (Callosciurus erythraeus) was introduced in Japan in the 1930s and has since established itself in several areas across the country. Although wild Sciuridae populations have been demonstrated to be potential reservoirs for zoonotic enteric protozoa, epidemiological studies of such pathogens in Japan are scarce. Here, we examined 423 fecal samples from Pallas’s squirrels captured in Kanagawa Prefecture, Japan, using PCR and DNA sequencing to determine the occurrence of *Cryptosporidium* spp., Enterocytozoon bieneusi, and *Blastocystis*. The overall prevalence of *Cryptosporidium* spp., E. bieneusi, and *Blastocystis* was 4.3% (18/423 samples), 13.0% (55/423 samples), and 44.0% (186/423 samples), respectively. The prevalence of *Blastocystis* and E. bieneusi was significantly higher in spring (60.1% and 17.4%, respectively) than in winter (27.6% and 8.6%, respectively [*P < *0.01]). Sequence analysis of *Cryptosporidium* spp., targeting the partial small subunit ribosomal RNA gene (SSU rDNA), showed 100% identity (541/541 bp) to Cryptosporidium ubiquitum, and analysis of the gp60 gene showed 99.76% (833/835 bp) identity to C. ubiquitum subtype XIIh. The sequences of the ribosomal internal transcribed spacer region of E. bieneusi and the partial SSU rDNA of *Blastocystis* were identified as E. bieneusi genotype SCC-2 and *Blastocystis* subtype 4, respectively. This study confirmed the presence of C. ubiquitum, E. bieneusi, and *Blastocystis* in Pallas’s squirrels in Kanagawa Prefecture. Because Pallas’s squirrels inhabit urban areas, living close to humans, the species may serve as a potential source of infection in human populations.

**IMPORTANCE** Pallas’s squirrel is designated a “regulated organism” under the Invasive Alien Species Act in Japan, and municipal authorities are introducing control measures to reduce its populations. It has been suggested that wild mammals may play a role in contaminating the environment with zoonotic pathogens. The present study detected the enteric pathogens Cryptosporidium ubiquitum, Enterocytozoon bieneusi, and *Blastocystis* in the feces of Pallas’s squirrels inhabiting Kanagawa Prefecture, Japan. These pathogens persist in the environment and contaminate soils and water, which may potentially infect humans. Because Pallas’s squirrels in Kanagawa Prefecture are found in urban areas, where they are in close contact with human populations, continued monitoring of zoonotic diseases among squirrel populations will be important for evaluating the significance of wildlife in pathogen transmission.

## INTRODUCTION

Pallas’s squirrel (Callosciurus erythraeus), or red-bellied squirrel, is a species native to China, India, and Southeast Asia. It has also established itself in several other countries, including Japan, Argentina, France, and the Netherlands (1). In Japan, the species is suggested to have been introduced from Taiwan to Izu Oshima Island in the 1930s and subsequently transferred to other parts of Japan. Damage to forest plantations, agricultural crops, and housing has been reported where the species is established, and the Japanese Ministry of the Environment has designated the species a “regulated organism” under the Invasive Alien Species Act (https://www.env.go.jp/en/nature/as.html).

*Cryptosporidium* spp., Enterocytozoon bieneusi, and *Blastocystis* are enteric protozoa, all of which have been reported in wild Sciuridae populations in the United States, Europe, and China (Table 1). Among these parasites, several zoonotic species/genotypes, such as C. parvum, E. bieneusi genotype IV, and E. bieneusi genotype D, have been detected and could potentially affect human health, especially in immunocompromised populations (2). The infectious stage of these pathogens is environmentally resistant and has been detected on water surfaces and agricultural products (3), causing outbreaks in the human population (4, 5). These pathogens can also infect wildlife, which then results in shedding of the pathogens from infected animals and subsequent contamination of the environment, serving as a potential source of infection for the human population. The same species and genotypes of *Cryptosporidium* spp. and E. bieneusi were detected in the feces of wild mammals and water sources where wildlife lives, suggesting a significant role of wildlife in pathogen contamination (6, 7).

**TABLE 1:** *Cryptosporidium* spp., Enterocytozoon bieneusi, and *Blastocystis* previously identified by molecular methods in wild tree squirrels (*Callosciurus*, *Sciurus*, and *Tamiasciurus*)

Pathogen and reported host	Species/genotype	Country	Reported in humans	Reference
*Cryptosporidium* spp.				
American red squirrel (Tamiasciurus hudsonicus)	C. ubiquitum XIIb	USA	Yes	39, 40
	Chipmunk genotype I	USA	Yes (emerging)	39
	Deer mouse genotype III	USA	None to date	40
	Skunk genotype	USA	Yes	40
Eastern grey squirrel (Sciurus carolinensis)	*C.* muris	USA	Yes	6
	C. parvum	USA	Yes (major)	6, 39
	C. ubiquitum XIIb/XIIc/XIId	USA/Italy	Yes	6, 16, 39, 40
	Chipmunk genotype I	USA/Italy	Yes (emerging)	6, 16
	Deer mouse genotype III	USA	None to date	6, 40
	Skunk genotype	USA/Italy	Yes	6, 16, 39, 40
Eurasian red squirrel (Sciurus vulgaris)	C. ubiquitum XIId	USA	Yes	6
	Chipmunk genotype I	Italy	Yes (emerging)	41
	Ferret genotype	Italy	None to date	16, 41
Fox squirrel (Sciurus niger)	C. ubiquitum XIIc	USA	Yes	40
	Skunk genotype	USA	Yes	40
Pallas's squirrel (*Callosciurus erythraeus*)	Chipmunk genotype I	Italy	Yes (emerging)	16
Enterocytozoon bieneusi				
Eastern grey squirrel (Sciurus carolinensis)	Type IV (group 1)	USA	Yes	7
	WL4 (group 3)	USA	None to date	7
	WW6 (group 4)	USA	None to date	7
	PtEBV + WL21 (group 1)	USA	None to date	7
Pallas's squirrel (Callosciurus erythraeus)	D	China	Yes	42
*Blastocystis*				
Eurasian red squirrel (Sciurus vulgaris)	ST4	UK	Yes	27

In Kanagawa Prefecture, squirrel species have invaded fragmented forests and parks in urban areas, living close to humans, with municipal authorities introducing measures to control their populations. Wild Sciuridae populations in other parts of the world have already been demonstrated to be potential reservoirs for zoonotic enteric protozoa (Table 1). Pallas’s squirrel in Kanagawa may also play a crucial role in parasite transmission, posing a risk to public health. Here, we aimed to determine the occurrence and molecular characteristics of *Cryptosporidium* spp., E. bieneusi, and *Blastocystis* in Pallas’s squirrel from Kanagawa, Japan, and to evaluate the significance of these pathogens from a public health perspective.

## RESULTS

The overall prevalence of *Cryptosporidium* spp., E. bieneusi, and *Blastocystis* was 4.3% (18/423 samples), 13.0% (55/423 samples), and 44.0% (186/423 samples), respectively (Table 2). The prevalence of *Blastocystis* was significantly higher in spring (60.1%) than in winter (27.6%; *x*^2^ = 45.266 [*P < *0.01]). This difference in season also affected the prevalence of E. bieneusi, which was higher in spring (17.4%) than in winter (8.6%; *x*^2^ = 7.2384 [*P < *0.01]). The prevalence of *Cryptosporidium* spp. was not affected by season. Neither sex nor maturity had a significant effect on the prevalence of the three protozoa examined here. Coinfections were observed in 22 samples; 6 were infected with both *Cryptosporidium* spp. and *Blastocystis*, and 16 were infected with both E. bieneusi and *Blastocystis*. No squirrel was coinfected with all three protozoa.

**TABLE 2:** Overall prevalence and prevalence by season, sex, and maturity for Cryptosporidium ubiquitum, Enterocytozoon bieneusi, and *Blastocystis*

Organism	Total (*n *= 423)	Season		Sex		Maturity	
		Winter (*n *= 210)	Spring (*n *= 213)	Female (*n *= 170)	Male (*n *= 253)	Juvenile (*n *= 101)	Adult (*n *= 320)
Cryptosporidium ubiquitum ^*a*^							
No. (%)	18 (4.3)	6 (2.9)	12 (5.6)	5 (2.9)	13 (5.1)	5 (5.0)	13 (4.1)
*x*^2^		2.001 (*P *> 0.05)		1.2058 (*P *> 0.05)		2.5369 (*P *> 0.05)	
Enterocytozoon bieneusi							
No. (%)	55 (13.0)	18 (8.6)	37 (17.4)	29 (17.1)	26 (10.3)	19 (18.8)	36 (11.3)
*x*^2^		7.2384 (*P* < 0.01)		4.1346 (*P *> 0.05)		3.8652 (*P *> 0.05)	
*Blastocystis*							
No. (%)	186 (44.0)	58 (27.6)	128 (60.1)	77 (45.3)	109 (43.1)	42 (41.6)	144 (45.0)
*x*^2^		45.266 (*P *< 0.01)		0.2018 (*P *> 0.05)		0.3632 (*P *> 0.05)	

a Samples were determined to be positive when sequencing results for the small subunit ribosomal RNA gene were identified as C. ubiquitum.

**FIG 1:**
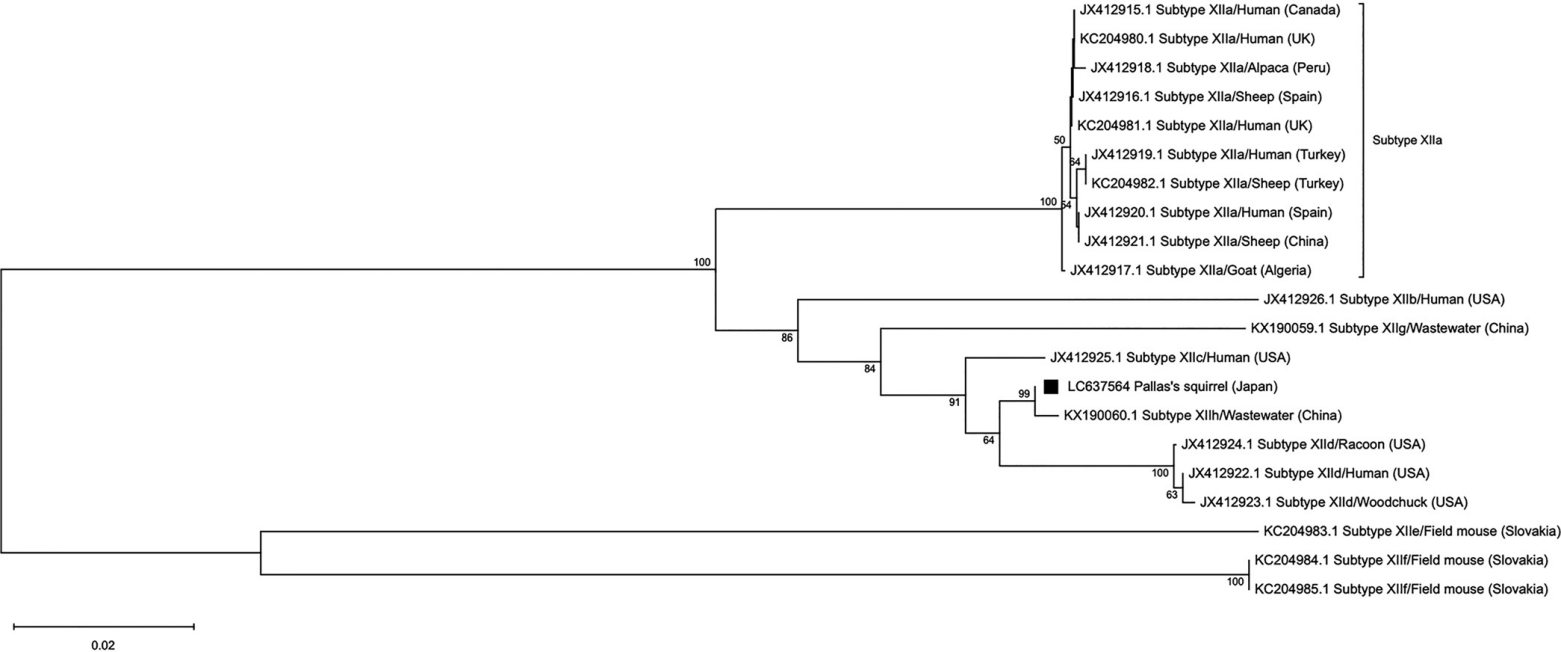
Phylogenetic relationship of Cryptosporidium ubiquitum subtypes inferred from gp60 gene sequences using the neighbor-joining method, based on the evolutionary distances calculated by the Kimura two-parameter model. The consensus tree was obtained after bootstrap analysis with 1,000 replications, with values of more than 50% shown in the tree. The tree is drawn to scale, with branch lengths measured in the number of substitutions per site. The analyses involved 21 nucleotide sequences and were conducted in MEGA X. Each sequence is identified by its GenBank accession number, subtype, and host origin. The sequence of the gp60 gene from Pallas’s squirrel in the present study is indicated by a black square.

Sequence analysis of the *Cryptosporidium* sp. partial small subunit ribosomal RNA gene (SSU rDNA) was successful for all 18 samples and showed 100% identity (541/541 bp) to the standard sequence for C. ubiquitum (GenBank accession number HM209366). PCR targeting the actin gene yielded positive results for 16 samples, and a 953-bp fragment was successfully sequenced, showing 100% identity (945/945 bp) to C. ubiquitum isolated from the Eastern gray squirrel (Sciurus carolinensis) (GenBank accession number KT027499) and the American red squirrel (Tamiasciurus hudsonicus) (GenBank accession number KT027504) from the United States. Samples positive for SSU rDNA were further analyzed by using the gp60 gene for subtyping, and 14 samples were successfully sequenced. All sequences showed 99.76% identity (833/835 bp) to C. ubiquitum subtype XIIh isolated from wastewater samples (GenBank accession number KX190060). The phylogenetic relationships of the C. ubiquitum subtype families to the sequences obtained in this study are described in Fig. 1.

**FIG 2:**
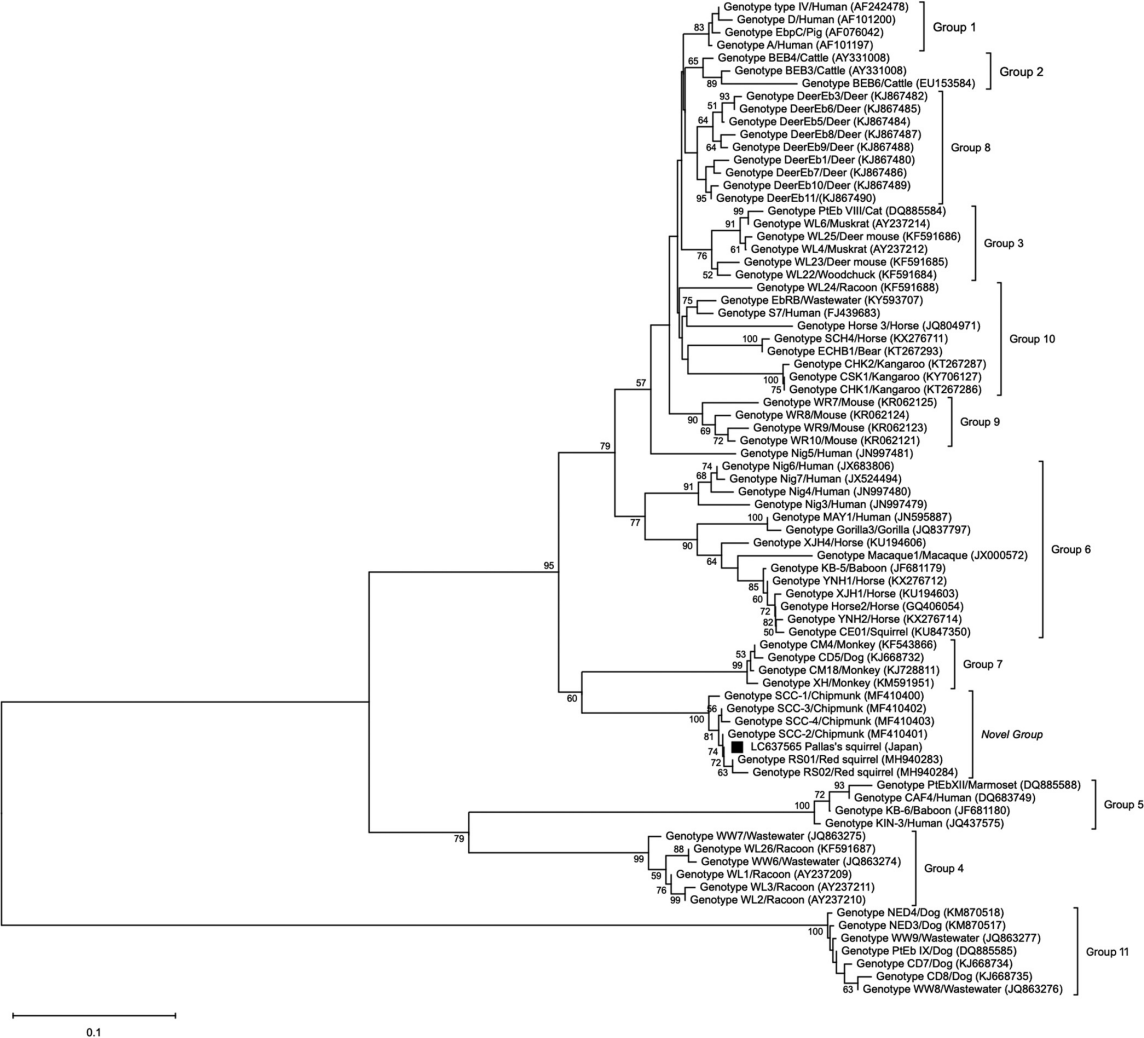
Phylogenetic relationship of Enterocytozoon bieneusi genotypes inferred from ITS gene sequences using the neighbor-joining method, based on the evolutionary distances calculated by the Kimura two-parameter model. The consensus tree was obtained after bootstrap analysis with 1,000 replications, with values of more than 50% shown in the tree. The tree is drawn to scale, with branch lengths measured in the number of substitutions per site. The analyses involved 78 nucleotide sequences and were conducted in MEGA X. Each sequence is identified by its genotype, host origin and GenBank accesion number in parentheses. The sequence of the E. bieneusi ITS gene from Pallas’s squirrel in the present study is indicated by a black square.

Sequence analysis of the E. bieneusi internal transcribed spacer (ITS) region was successful for all 55 samples, and all sequences showed 100% identity to E. bieneusi genotype SCC-2, which was isolated from the feces of the chipmunk *Eutamias asiaticus* from China (GenBank accession number MF410401). Phylogenetic analysis of the ITS gene sequences revealed that genotype SCC-2 clustered in a separate group distinct from the 11 known validated groups (Fig. 2).

Of the 186 samples that were positive for *Blastocystis*, 85 were subjected to sequence analysis. The results for the SSU rDNA sequencing for all samples were identical to each other and identified as sequence type 4 (ST4).

## DISCUSSION

In this study, we revealed for the first time the presence of C. ubiquitum, E. bieneusi, and *Blastocystis* in Pallas’s squirrel populations in Kanagawa Prefecture. The habitat distribution of Pallas’s squirrel in Kanagawa Prefecture is in fragmented woodlands located between residential and industrial areas, where human activities are in close proximity (8). This indicates the potential of Pallas’s squirrel to contaminate the environment and subsequently to serve as a reservoir for C. ubiquitum, E. bieneusi, and *Blastocystis*, leading to human infection.

C. ubiquitum was previously known as the *Cryptosporidium* cervine genotype and has emerged as a significant zoonotic species, primarily in industrialized nations (9). The species has been found in a broad range of mammalian species, including ruminants, rodents, and carnivores, and has also been detected in various water samples (9–11). In Japan, only two studies reporting C. ubiquitum infections have been published to date, one from wild Japanese field mice (Apodemus speciosus) and another from imported pet chinchillas (Chinchilla lanigera) (12, 13). Currently, eight subtypes, XIIa to XIIh, have been identified for C. ubiquitum using the gp60 gene (14, 15). The distribution of these subtypes is suggested to be determined by host species and geographical locations. Subtype XIIa has been found in ruminant hosts worldwide. Subtypes XIIb to XIId have been found in rodent hosts, including wild tree squirrels, in North America (Table 1), and subtypes XIIe and XIIf have been found in field mice in the Slovak Republic (14). Confirmed zoonotic subtypes are XIIa to XIId, which have been reported mainly in the United States and the United Kingdom (14). Genotype XIId has also been reported in Eastern gray squirrels in Italy, indicating the possibility that this genotype was introduced to Europe from North America together with Eastern gray squirrels (16). The latest C. ubiquitum genotypes reported are XIIg and XIIh, from wastewater in China, which clustered with the rodent subtype families XIIb to XIId, rather than with ruminant-adapted subtype XIIa (15). In the present study, we identified genotype XIIh from rodent hosts, which further suggests that this genotype is indeed rodent adapted and also indicates that Pallas’s squirrel is a potential source of environmental contamination for this genotype.

E. bieneusi has been reported in a wide range of domestic and wild animals and is the most common microsporidian species reported in humans (17, 18). Up to 474 distinct genotypes have been identified in both humans and animals worldwide by phylogenetic analysis of ITS nucleotide sequences, which cluster in 11 major genetic groups (19). Genotypes that belong to groups 1 and 2 have a broad host range, including humans, whereas those in groups 3 to 11 are more host specific (19). The genotype SCC-2 identified here was found first in pet chipmunks (*Eutamias asiaticus*) and then in pet red squirrels (Sciurus vulgaris), both reported in China (20, 21). This genotype did not belong to any of the 11 validated genotypes but formed a separate cluster together with other genotypes found in the Sciuridae family (Fig. 2). It is not yet known whether the host range of this genotype is limited to rodents, specifically Sciuridae, or involves other host species, including humans. In Japan, the presence of zoonotic E. bieneusi, including genotypes K, D, and EbpC, has been reported in domestic cats, dogs, and pigs, but there are no reports of clinical cases in humans (22, 23). Although the significance of E. bieneusi infection may be low in Japan, it is important to recognize the potential role of Pallas’s squirrel in maintaining the life cycle of the parasite.

The prevalence of *Blastocystis* was the highest among the parasites examined here, indicating that *Blastocystis* infection is common in Pallas’s squirrels from Kanagawa, irrespective of sex or maturity. Currently, *Blastocystis* isolates from mammalian and avian hosts are classified into 17 STs, 10 of which (ST1 to ST9 and ST12) have been reported in humans (24, 25). ST4 is the only ST identified in this study and is a major subtype found in wild rodents, including brown rats (Rattus norvegicus), Polynesian rats (Rattus exulans), red squirrels, and water voles (Arvicola amphibius), demonstrating strong host adaptation of this ST (26–29). Although ST4 from mammals is frequently reported worldwide, the geographical distribution of ST4 in the human population is mostly limited to European countries (24). A study in an Indonesian community reported ST4 in Polynesian rats but not in human or other animal populations in the community. This indicates that transmission of *Blastocystis* to human populations from rodent species may not occur frequently (26).

In the present study, we observed significant seasonal differences for E. bieneusi and *Blastocystis*, with higher prevalence in spring than in winter. The activities of Pallas’s squirrel in Japan are reported to decrease significantly when the temperature falls during winter months, compared to squirrels in their native habitats in subtropical climate zones (30). This decrease in activity might have resulted in less contact with other squirrels, resulting in less chance of parasite transmission during the winter months. Moreover, the mating period for squirrels peaks between March and August (31), resulting in increased contact between males and females during these warmer months. Another key indicator for determining the risk of transmission is estimating the environmental loading of parasites from squirrels. For instance, fecal oocyst shedding of C. parvum from California ground squirrels (Spermophilus beecheyi) fluctuated throughout the year, coinciding with changes in the host’s population structure (32). Infected dams can transmit the pathogen to newborns, which have higher levels of oocyst shedding due to their immature immune status. These juveniles emerge from their burrows in May, resulting in greater environmental loading and greater prevalence among other squirrels, including mature individuals (32). Neither sex nor maturity had a significant effect on the prevalence of the parasites examined here; however, it is possible that this is not the case for fecal shedding intensity. Depending on the quantity of shedding and the fact that Pallas’s squirrel has high population density, with reproduction occurring throughout the year (31), environmental loading may have a significant impact on the transmission of C. ubiquitum, E. bieneusi, and *Blastocystis*. It is crucial to integrate the ecology of Pallas’s squirrel and to make an effort to evaluate the environmental loading of pathogens in future studies.

This study confirmed the presence of C. ubiquitum, E. bieneusi, and *Blastocystis* in Pallas’s squirrel in Kanagawa Prefecture, Japan. The presence of these enteric pathogens confirms the need for long-term monitoring of zoonotic diseases in squirrel populations. Such information will help us better understand the role of wildlife in parasite transmission and the risk to public health. Consequently, such information will enable the establishment of effective control strategies where necessary.

## MATERIALS AND METHODS

### Study site and sample collection.

A total of 423 Pallas’s squirrels were captured using single-capture live traps, which were set overnight at three locations within a 3-km radius in the city of Hayama on Miura Peninsula, Kanagawa Prefecture, Japan. Trapping was approved by Hayama city and conducted by EGO Co., Ltd. (Kanagawa, Japan). Trapping took place between January and June in 2018 to 2020. The sample numbers for 2018, 2019, and 2020 were 131, 124, and 168, respectively. Trappings were conducted for 3 to 5 consecutive days each month, with traps baited with peanuts and sesame oil. Captured squirrels were euthanized at the trapping site by sevoflurane inhalation and were kept at −30°C until use. The sex, body weight, and maturity of the animals were recorded, and fecal samples were collected from the rectum upon dissection. The maturity of the animals was determined on site by scrotal and nipple pigmentation for males and females, respectively.

### DNA extraction, PCR, and sequencing.

Genomic DNA was extracted from 423 fecal samples using the QIAamp Fast DNA stool minikit (Qiagen, USA) according to the manufacturer’s protocol. Fecal samples were homogenized in InhibitEX buffer (included in the kit) using bead tubes (AMS Co., Ltd., Japan) for the destruction of E. bieneusi spores (33). *Cryptosporidium* spp. were identified using primers that amplify partial SSU rDNA (34). Samples that were positive with these primers were further analyzed using the actin gene (35) and the 60-kDa glycoprotein (gp60) gene (14) for species confirmation and genotyping. E. bieneusi was identified using primers that amplify the entire ribosomal ITS region (–243 bp) (36). *Blastocystis* was identified by partial (320 to 342 bp) SSU rDNA using primers BL18SPPF1 and BL18SR2PP (37). The primers used are shown in Table 3. PCR was performed in a 50-μl volume, including 1 μl of DNA template, 1× *Ex Taq* buffer, 1.25 U of *Ex Taq* DNA polymerase (TaKaRa Bio Inc., Japan), 0.2 mM deoxynucleoside triphosphate (dNTP) mixture, and 0.2 μM primers. The cycling conditions for each primer were determined as described previously (14, 34–37). Amplified products were visualized on a 1.0% to 1.5% agarose gel. Samples were determined to be positive when a specific band was visible in the gel. Positive PCR products were purified using the NucleoSpin gel and PCR clean-up kit (Macherey-Nagel, Germany) according to the manufacturer’s protocol and were directly sequenced on both strands using the same primers as used for the PCR, in a sequencing facility (FASMAC Co., Ltd., Japan).

**TABLE 3:** Primers for *Cryptosporidium* spp., Enterocytozoon bieneusi, and *Blastocystis* used in this study

Organism and target gene	Primers	Sequences	Size (bp)	Reference
*Cryptosporidium* spp.				
SSU rDNA	Primary (18SiCF2 and 18SiCR2)	5′-GACATATCATTCAAGTTTCTGACC-3′ and 5′-CTGAAGGAGTAAGGAACAACC-3′	763	34
	Secondary (18SiCF1 and 18SiCR1)	5′-CCTATCAGCTTTAGACGGTAGG-3′ and 5′-TCTAAGAATTTCACCTCTGACTG-3′	∼587	
Actin	Primary	5′-ATG(A/G)G(A/T)GAAGAAG(A/T)A(A/G)(C/T)(A/T)CAAGC-3′ and 5′-AGAA(G/A)CA(C/T)TTTCTGTG(T/G)ACAAT-3′	∼1,095	35
	Secondary	5′-CAAGC(A/T)TT(G/A)GTTGTTGA(T/C)AA-3′ and 5′-TTTCTGTG(T/G)ACAAT(A/T)(G/C)(A/T)TGG-3′	∼1,066	
gp60	Primary (Ubi-18S-F1 and Ubi-18S-R1)	5′-TTTACCCACACATCTGTAGCGTCG-3′ and 5′-ACGGACGGAATGATGTATCTGA-3′	1,044	14
	Secondary (Ubi-18S-F2 and Ubi-18S-R2)	5′-ATAGGTGATAATTAGTCAGTCTTTAAT-3′ and 5′-TCCAAAAGCGGCTGAGTCAGCATC-3′	948	
E. bieneusi				
ITS	Primary (EBITS3 and EBITS4)	5′-GGTCATAGGGATGAAGAG-3′ and 5′-TTCGAGTTCTTTCGCGCTC-3′	435	36
	Secondary (EBITS1 and EBITS2.4)	5′-GCTCTGAATATCTATGGCT-3′ and 5′-ATCGCCGACGGATCCAAGTG-3′	390	
*Blastocystis*				
SSU rDNA	Partial	5′-AGTAGTCATACGCTCGTCTCAAA-3′ and 5′-TCTTCGTTACCCGTTACTGC-3′	320–342	37

### Phylogenetic analysis.

Sequences were compared with those of *Cryptosporidium* spp., E. bieneusi, and *Blastocystis* homologous sequences available from GenBank (National Center for Biotechnology Information) using the nucleotide Basic Local Alignment Search Tool (BLAST) program (https://blast.ncbi.nlm.nih.gov/Blast.cgi). Species and/or genotypes of examined protozoa were determined by an exact match or identity of ≥98% against all known species or genotypes found in mammals and birds, with a query coverage of ≥98%. Phylogenetic analyses were conducted to clarify the relationship between the genotypes of *Cryptosporidium* spp. and E. bieneusi. Sequences obtained in this study were aligned with reference sequences available from GenBank using ClustalW implemented in MEGA X (38). Analyses were performed using the neighbor-joining method based on the evolutionary distances calculated by the Kimura two-parameter model implemented in MEGA X (38). The reliability of these trees was assessed by bootstrap analysis with 1,000 replicates.

### Statistical analysis.

The prevalence of each enteric protozoan according to sex (female versus male), maturity (adult versus juvenile), and season (winter [January to March] versus spring [April to June]) was analyzed using the chi-square test for independence. Seasons were defined by the monthly average temperature for the Miura Peninsula between 2018 and 2020, as provided by the Japan Meteorological Agency database (https://www.data.jma.go.jp/obd/stats/etrn/index.php) (in Japanese). The monthly average temperatures in January, February, and March from 2018 to 2020 were 6.1 to 8.0°C, 6.3 to 8.0°C, and 11.1 to 11.9°C, respectively, and the temperature did not exceed 10°C most days. The average monthly temperature from April to June was above 13.5°C for all of the years examined. Statistical differences with *P* values of <0.05 were considered significant.

### Data availability.

The sequences obtained in this study were deposited in GenBank under the accession numbers LC637562 to LC637566.
